# General Commentary: Whether Urbanization Has Intensified the Spread of Infectious Diseases—Renewed Question by the COVID-19 Pandemic: Urban Density and COVID-19: Correlation Between Densification, Unhealthy Urban Conditions, and the Spread of Infectious Diseases

**DOI:** 10.3389/fpubh.2022.917350

**Published:** 2022-07-13

**Authors:** Ali Cheshmehzangi, Tong Zou, Zhaohui Su, Ayotunde Dawodu

**Affiliations:** ^1^Network for Education and Research on Peace and Sustainability (NERPS), Hiroshima University, Hiroshima, Japan; ^2^Department of Architecture and Built Environment, University of Nottingham Ningbo China, Ningbo, China; ^3^School of Public Health, Southeast University, Nanjing, China

**Keywords:** urban density, COVID-19, densification trend, unhealthy urban conditions, disease spread, public health, cities

The ongoing COVID-19 pandemic attracts numerous research interests across various fields and disciplines. Since most populations live in the cities, research in public health and urban studies regarding pandemic prevention and control in higher-density areas has become one of the top priorities in this new pandemic era. Studies revealed that population urbanization-introduced density growth does not increase the spread of infectious disease, while land urbanization-introduced building density growth can increase the risks of spreading infectious diseases (1). Moreover, regional heterogeneity is found in the distribution of impacts of urbanization on the spread of infectious diseases (ibid). Yu et al. (1) found that greater population urbanization can reduce the morbidity, mortality, and spread of class A and B infectious diseases, while greater land urbanization has the opposite impact. In China, Class A only contains plague and cholera, and class B includes SARS, AIDS, anthrax, tuberculosis, typhoid, etc. (2). Coronavirus pneumonia belongs to class B infectious disease in China. It has been treated as class A due to its high risks of spreading strong infectivity and wide range of spreading. Thus, those findings may also be applied to the scenarios of COVID-19 outbreaks in China's urban areas to some extent.

In Yu's et al. (1) study, population urbanization is determined by the proportion of the urban population, while the density of urban buildings measures land urbanization. Such consideration may not fully reflect the actual level and degree of the two types of urbanization. Also, another critical determining factor of spreading infectious diseases related to population density should be considered as people's mobility. This factor is related to the infectious source and/or virus needed to be transmitted in the first place to increase the risk of infecting others and spreading widely. However, China has adopted stringent and different COVID-19 pandemic control and prevention policies, different from other countries. This approach is widely known as the zero-COVID approach or zero-tolerance concept (3). As a consequence of this zero-COVID policy, people living in areas with higher urban density may be required to stay at home for quarantine and do regular testing during a specific time. In some cases, like in Shanghai's recent outbreak in March 2022, some people are relocated to local government-assigned quarantine locations (e.g., cabin hospitals and hotels) once one or multiple infected cases are found, or lockdown is imposed.

Moreover, regarding context specificity, not only do countries have different pandemic control and prevention policies and protocols, but also provinces and cities in China have different interpretations and implementations of the central government policies. Such a decentralized approach varies in making regional guidelines and governance. For instance, we noticed differences in the case of Shanghai in April 2022 and Wuhan in January 2020. Regarding those points, the conclusion drawn from China's provincial and city-level data without consideration of mobility may need more consideration and comprehensive investigations covering the factors related to people's mobility under different unique pandemic control and prevention strategies such as city-wide lockdown, health code illustrating health code, and durations of home quarantines.

To date, scholarly research studies that only focus on the correlation between density and COVID-19 spread do not address the main issues related to urban density. They often neglect the combined effect between density and other negativities (4), such as hygiene or sanitation issues, socio-economic disparities, overcrowding, higher connectivity, higher mobility, spatial conditions, poverty, etc. Density is often entangled with other urban geographies, suggesting how it can couple with other factors and become a major urban problem (5). Therefore, it is not accurate to jump to immediate conclusions that “*population density has no obvious impact on the spread of infectious diseases*” (1). In fact, any disease spread could be intensified with higher population density, arguing in favor of the point that unplanned urban densification could lead to unhealthy urban conditions; and hence, faster spread of infectious diseases. This viewpoint cannot be supported when density alone is studied, and spatio-temporal studies are not accurate.

We urge ongoing research to pay more attention to negativities associated with urban density. Such studies should consider multiple factors for the correlation analysis of density issues. Scholarly research must not only explore a linear correlation between two factors of density and disease spread and should instead understand the other contextual factors and urban characteristics that may be influential in intensifying the negative impacts of high-density urban patterns.

Here, we provide a few examples of how the correlation between population density and influential negativities (4, 6, 7) matters more than just density alone. When it comes to poorer communities, there are issues related to intergenerational living, higher occupation risks, potential unsanitary issues, and lower socio-economic levels (8–10). Hence, a dense poor community could contain many risks for disease spread. Concerning land-use planning issues, a balance is needed between mixed-use development, density, and compactness of the urban environments. There are higher risks of population density and unnecessary mobility in the mono-functional compact urban layouts, which could also relate to urban morphologies (i.e., urban form and configuration). Another example is related to overcrowding issues, often linked with less efficient critical infrastructures in dense urban environments (11–13). Poorer urban services could lead to lower-level urban public health and safety, which means dense urban areas may have little room for diverse and efficient urban services, such as good quality healthcare services, public transportation networks, food delivery and distribution systems, etc. Lastly, we refer to small-scale matters or details of the built environments, such as micro-level factors like ventilation, waste management, housing quality, provisions, and attributes, social services, etc. These factors often perform lower or are minimized in dense urban areas.

In sum, we urge researchers to refrain from singular correlation analysis between density and disease spread. Scholarly research in this area must be accurate and comprehensive to ensure the right data is used, analysis is conducted flawlessly, and correlations are complex and valid. Therefore, density mattes, in combination with negativities and disease spread. We suggest future research include and evaluate context-specific factors. This should be done more carefully to avoid one-size-fits-all solutions and/or suggestions. More importantly, we anticipate future policies and reforms to respond to density matters. Future urban planning and design paradigms should be aligned with healthy city indicators and consider urban public health as a significant driver to creating better living and working environments.

## Author Contributions

AC drafted the idea and paper narrative. TZ helped with structure and paper development. ZS and AD worked on reviews and revision. All authors contributed to the article and approved the submitted version.

## Funding

AC acknowledges the National Natural Science Foundation of China (NSFC) for the provision of funding for project number 71950410760. He also acknowledges the Ministry of Education, Culture, Sports, Science and Technology (MEXT), Japan Government, and the Network for Education and Research on Peace and Sustainability (NERPS), Hiroshima, Japan.

## Conflict of Interest

The authors declare that the research was conducted in the absence of any commercial or financial relationships that could be construed as a potential conflict of interest.

## Publisher's Note

All claims expressed in this article are solely those of the authors and do not necessarily represent those of their affiliated organizations, or those of the publisher, the editors and the reviewers. Any product that may be evaluated in this article, or claim that may be made by its manufacturer, is not guaranteed or endorsed by the publisher.
